# Continuous and Non-Invasive Lactate Monitoring Techniques in Critical Care Patients

**DOI:** 10.3390/bios14030148

**Published:** 2024-03-18

**Authors:** Jose-Luis Lafuente, Samuel González, Clara Aibar, Desirée Rivera, Eva Avilés, Juan-Jose Beunza

**Affiliations:** 1 IASalud, Universidad Europea de Madrid, Villaviciosa de Odón, 28670 Madrid, Spain; joseluis.lafuente@universidadeuropea.es (J.-L.L.); samuel.gonzalez@grupohla.com (S.G.); aibar.claraalv@gmail.com (C.A.); desiree.rivera.rdz@gmail.com (D.R.); eva.aviles@universidadeuropea.es (E.A.); 2 Engineering Department, School of Architecture, Engineering & Design, Universidad Europea de Madrid, Villaviciosa de Odón, 28670 Madrid, Spain; 3 Intensive Care Unit, Hospital Universitario HLA Moncloa, 28008 Madrid, Spain; 4 Research and Doctorate School, Universidad Europea de Madrid, Villaviciosa de Odón, 28670 Madrid, Spain; 5 Department of Medicine, Health and Sports, Universidad Europea de Madrid, Villaviciosa de Odón, 28670 Madrid, Spain

**Keywords:** lactate, biosensors, non-invasive sensors, monitoring in critical care environments, sensor technologies, IoMT

## Abstract

Lactate, once merely regarded as an indicator of tissue hypoxia and muscular fatigue, has now gained prominence as a pivotal biomarker across various medical disciplines. Recent research has unveiled its critical role as a high-value prognostic marker in critical care medicine. The current practice of lactate detection involves periodic blood sampling. This approach is invasive and confined to measurements at six-hour intervals, leading to resource expenditure, time consumption, and patient discomfort. This review addresses non-invasive sensors that enable continuous monitoring of lactate in critical care patients. After the introduction, it discusses the iontophoresis system, followed by a description of the structural materials that are universally employed to create an interface between the integumentary system and the sensor. Subsequently, each method is detailed according to its physical principle, outlining its advantages, limitations, and pertinent aspects. The study concludes with a discussion and conclusions, aiming at the design of an intelligent sensor (Internet of Medical Things or IoMT) to facilitate continuous lactate monitoring and enhance the clinical decision-making support system in critical care medicine.

## 1. Introduction

Lactate is a metabolite present in blood composition as well as in a majority of bio-fluids such as sweat, saliva, and interstitial fluid. Within human physiology, lactate is directly produced in its ionized form during glucose metabolism, predominantly comprising L-lactate, which is the physiologically more significant isomer. While often erroneously associated with lactic acid, lactate is in fact an anion and a conjugate base, whereas lactic acid (C_3_CH(OH)COOH) dissociates into lactate (C_3_CH(OH)COO^−^) and protons (H^+^), with the latter contributing to acidification. Lactate production occurs under both aerobic and predominantly anaerobic conditions. In aerobic conditions, pyruvate generated from glycolysis enters the Krebs cycle, significantly mitigating lactate production. Under anaerobic circumstances, lactate serves as a final product of glycolysis and participates in the Cori cycle for gluconeogenesis [1,2,3].

Lactate has emerged as a pivotal metabolite in human biochemistry, garnering recognition as a critical clinical marker across various medical disciplines. Historically perceived as a byproduct of anaerobic metabolism, its role in human physiology and pathology is increasingly acknowledged as complex and enlightening [4,5,6]. Once solely associated with hypoxia and muscular fatigue, key functions of lactate in cellular signaling, immune response, and blood pH regulation have now been unveiled. Recent research has debunked the notion of its limited role [7], repositioning lactate as an essential metabolite with significant implications for diagnosis and medical monitoring, spanning conditions such as septic shock, heart failure, and metabolic disorders [8].

Hyperlactatemia is defined as abnormally high lactate levels, typically exceeding 2 to 2.5 mM, often resulting from a confluence of factors including increased lactate production, decreased clearance, or tissue hypoperfusion. This lactate elevation is frequently linked with mitochondrial dysfunction, hypermetabolic stress, hepatic dysfunction, and shock states. Notably, in patients with sepsis and septic shock, lactate serves as a valuable marker for assessing tissue hypoperfusion and as an endpoint for resuscitation efforts.

The current methodology for lactate level detection involves obtaining a blood sample and subsequent analysis in a laboratory setting. The standardized time interval between such measurements is approximately six hours. This technique is notably invasive, as patients may undergo venipuncture at least four times within a 24 h period. The development of new techniques and the design of non-invasive continuous monitoring sensors would represent a significant advancement in the field of intensive care medicine, providing the potential for automated, ongoing responses.

Lactate is a critical prognostic biomarker in critical care medicine, indicative of disease severity and treatment efficacy. Its monitoring in Intensive Care Units (ICUs) is essential for therapeutic decision-making and improving patient outcomes [9]. Current guidelines for the care of patients with sepsis recommend lactate measurement in suspected cases and utilizing lactate levels to guide resuscitation maneuvers [10]. Evidence has shown that, during the initial 24 hours in the ICU, lactate clearance is the most reliable parameter associated with 28-day mortality rates in septic patients [11]. Therefore, it is suggested that treatment protocols be oriented towards lactate clearance in such patients, even beyond the initial critical hours, highlighting lactate’s role as a valuable tool in the assessment of critically ill patients. Additionally, in scenarios such as myocardial infarction, pulmonary embolism, trauma, or acute respiratory distress syndrome, among others, elevated lactate levels may also indicate adverse clinical outcomes and serve as predictors of mortality [1,2,3].

Consequently, it is essential to consider lactate as a multifaceted biomarker that provides insights into the metabolic status and severity of various clinical conditions. Owing to lactate’s rapid concentration fluctuations and its clinical significance, frequent measurements are imperative in patients requiring continuous monitoring. This necessity has spurred the development of point-of-care devices facilitating direct and swift measurements in medical settings. These devices are categorized into two types: benchtop analyzers and smaller, portable devices, with some specifically designed for lactate measurement. Regardless of the platform, blood sample extraction is typically performed invasively via an intravenous line and must be repeated for accurate lactate kinetics assessment [12]. Despite their advantages, these devices have limitations. The need for repeated measurements and its consequent negative impact on workload, along with the risk of iatrogenic anemia in patients, has underscored the urgency for developing non-invasive technologies for continuous and precise lactate monitoring in critical medical environments [12,13].

This demand has led to the advent of biosensors, which offer an innovative and promising alternative for non-invasive lactate measurement in these settings. Biosensors are devices that integrate specific biochemical reactions with detection systems, providing non-invasive methods for real-time continuous lactate monitoring, thereby overcoming the current limitations of point-of-care technologies. Given the rapid advancement of non-invasive lactate sensing technologies, it has become pertinent to delve into a detailed analysis of existing biosensor types, their current developmental state, and potential future evolutions.

**Figure 1:**
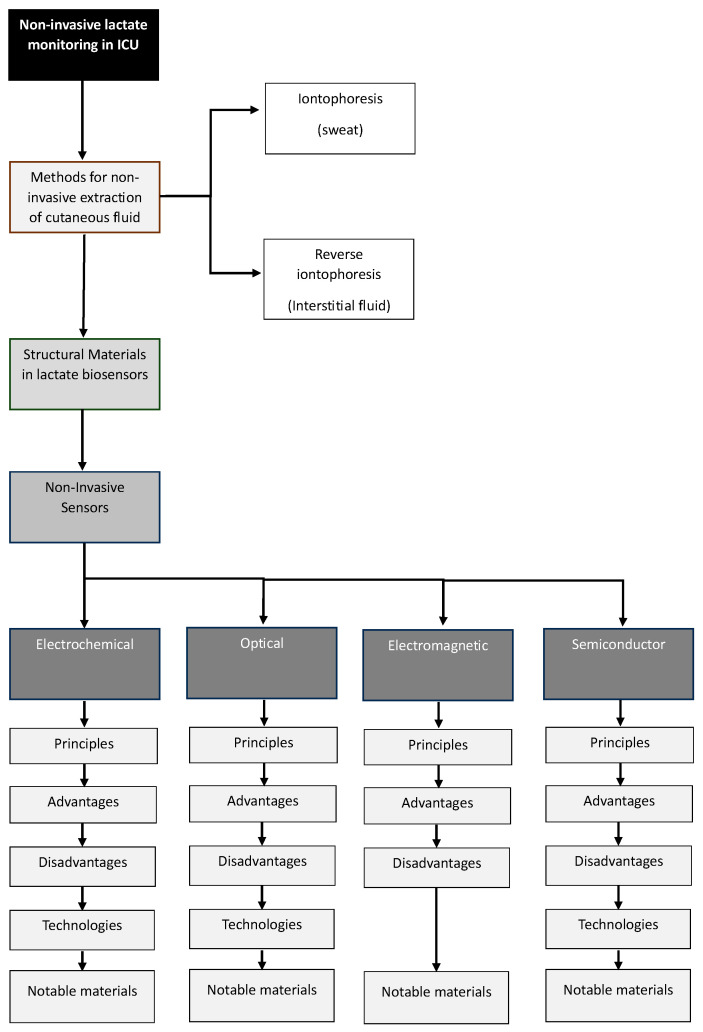
Organization of this study.

This study addresses, first of all, the development of non-invasive methods for fluid extraction with a focus on ICUs, centering on techniques such as iontophoresis and reverse iontophoresis. A section is dedicated to the analysis of structurally relevant materials in this context. The implementation of non-invasive sensors for continuous lactate monitoring is thoroughly examined, exploring different categories including electrochemical, optical, electromagnetic, and semiconductor-based sensors. Ultimately, this work aids in understanding the available tools and concludes by highlighting significant findings and emphasizing the importance of these innovations in the medical field, particularly in the continuous monitoring of critical patients to enhance the clinical decision-making support system. Figure 1 illustrates the general organization of this study.

## 2. Methods for Non-Invasive Extraction of Cutaneous Fluids: Iontophoresis and Reverse Iontophoresis

There are two prominent methods for the continuous measurement of variable lactate concentrations in biofluids: sweat measurement (iontophoresis) and interstitial fluid measurement (reverse iontophoresis).

Iontophoresis allows for the measurement of bodily lactate levels in sweat. These levels are typically significantly higher (5–40 mM) than those found in blood (0.5–2 mM). However, critically ill patients in ICUs usually do not release high volumes of sweat due to a lack of physical activity, making sweat collection difficult using this technique. Iontophoresis employs a low electrical current applied to the skin via electrodes, as well as the administration of a sweat-inducing agent (e.g., pilocarpine) through a hydrogel layer, stimulating the sweat glands. Sensors collect sweat samples for continuous biomarker monitoring [14,15,16]. The extraction method is non-invasive, painless, and noted for its practicality, simplicity, low cost, and portability, although potential skin burns or irritations are a concern. As sweating increases, the lactate concentration in sweat tends to decrease, which may complicate real-time monitoring and interpretation [14,15,16].

Reverse iontophoresis is based on the principles of electrochemistry and ion migration under the influence of an electric field. Interstitial fluid (IF or ISF) is an extracellular fluid present in the interstitium, around cells. It contains various markers, generally with concentrations in equilibrium with blood. Lactate is present in both the ISF and blood, with an approximate concentration of 1–2 mM in patients without alterations [17,18,19]. By applying an electric current in a controlled manner, the migration of charged molecules towards the biological membrane surface can be induced, facilitating their extraction. The dense and lipidic epidermis presents resistance to this process but allows the flow of ISF out of the dermal tissue [18]. The amount of ISF extracted varies according to the intensity and duration of the applied electric field [17,19]. This method enables the non-invasive extraction of molecules from the dermis, without causing tissue damage or requiring blood contact. Despite challenges associated with the anionic nature of lactate in the interstitial fluid, early measurement is achieved through high current and prolonged exposure. However, the risk of skin damage (e.g., burn) due to prolonged current application during extended exposure must be considered [17].

## 3. Structural Materials in Lactate Biosensors

Biosensors must fulfill key requirements to be efficient as an interface between the integumentary system and the sensor. Biocompatibility is crucial to prevent skin damage, necessitating chemical stability. Comfort is essential, favoring materials that are flexible, soft, light, adaptable, and elastic. Factors such as miniaturized design, portability, scalability, and cost are also critical considerations. This section describes the most relevant materials in the development of these types of biosensors.

### 3.1. Polyethylene Terephthalate (PET)

This polymer, widely used in textiles and films, is notable for its excellent mechanical properties and insulation. Its resistance to temperature and corrosion makes it an ideal material for manufacturing a flexible sensor. In its fabrication, a PET platform is used to screen-print electrodes, thus establishing stable contact between the sensor and the skin [20].

Researchers have developed lactate-monitoring biosensors utilizing PET’s exceptional properties. In a study by Alam et al. [21], a highly sensitive, selective, and flexible enzyme sensor was developed based on ZnO nanoflakes (ZnO-NFs) for non-invasive lactate detection in sweat. Here, gold was deposited on a flexible PET substrate as the sensor’s base. In another study by He et al. [22], a flexible patch capable of non-invasive, real-time monitoring of various biomarkers extracted from sweat, including lactate, was presented. In this case, the integration of different signals and data transmission was achieved using a digital laser processing technique to print circuits on a flexible PET substrate.

### 3.2. Paper

Paper, primarily composed of cellulose, excels among portable biosensors due to its low cost, biocompatibility, and sustainability. Its high surface-area-to-volume ratio enhances interaction with analytes, accelerates reactions, and increases detection capacity. Its structural properties, such as capillary absorption, facilitate the collection of microfluidic samples like sweat or saliva. Despite its utility, paper has limitations, including low sensitivity and difficulty performing the simultaneous detection of multiple analytes [20,23,24].

Paper stands out as a crucial support in identifying analytes through electrochemical properties, colorimetric changes, and light absorption. Acting as a matrix for electrodes, it provides a platform upon which samples and biomolecules bind and react. Its thin film, stabilized by fluids, transports analytes to the electrode surface. Paper fibers serve as a base for the deposition of redox materials and conductors, resulting in a significant increase in surface area and conductivity [23,24,25].

Vaquer et al. [26] developed a portable colorimetric optical biosensor to simultaneously measure lactate concentration and sample volume in sweat. Made with filter paper, this biosensor easily adhered to the skin and used gold nanoparticles to provide information about the sample volume, measuring the distance traveled by these nanoparticles transported by the paper. Xiao et al. developed a portable biocompatible paper-based biosensor that adheres to the skin and allows simultaneous in situ analysis of sweat pH and lactate. The paper used was characterized to prepare colorimetric sensors for the aforementioned analytes [27]. Additionally, Li et al. [28] developed a highly integrated sensing paper (HIS paper), involving low-cost, independent, and disposable detection for real-time sweat analysis. This paper combines hydrophobic protective wax, conductive electrodes, and MXene materials and was incorporated into an electrochemical biosensor that monitors glucose and lactate from sweat samples simultaneously.

Moreover, in a recent and pioneering study by Ruggeri et al. [29] a novel paper-based colorimetric sensor for continuous lactate monitoring in clinical settings is presented. This sensor offers rapid response, high precision, and sensitivity, along with a wide detection range. It stands out from previous studies due to its exceptional long-term stability, achieved using the silk fibroin matrix, which, when combined with the paper substrate, effectively protects against the degradation of enzymes and proteins during storage. Accelerated degradation tests demonstrate that the sensor maintains its detection capability and sensitivity even after exposure to extreme temperature conditions. After 2.5 months at 60 °C, it retained up to 66% of its activity, and after 24 months at 4 °C, it fully retained its activity. This unique combination makes it ideal for use in ICUs, where long-term stability is crucial for continuous monitoring.

### 3.3. Polydimethylsiloxane (PDMS)

This elastomeric polymer has interesting properties for biomedical applications, including physiological inertness, excellent resistance to biodegradation, biocompatibility, chemical stability, gas permeability, good mechanical properties including a low elastic modulus and high tensile properties, and excellent optical transparency. It is also easy to acquire, handle, and manipulate, and exhibits hyperelastic behavior [20,30].

Its primary application is seen in functional microfluidic devices where it is used for electronic encapsulation integrated on the surface or as a protective coating. In flexible biosensors, it is integrated with electrodes on the polymer surface [30,31]. This material was used in a study by Yokus et al. [32], which characterized a system for the continuous and non-invasive monitoring of lactate at low sweat rates. This portable biosensor consists of a hydrogel and paper-based microfluidic device with printed electrodes integrated on this polymer. In another study by Miesse et al. [33], the development of a non-invasive, portable electrochemical lactate biosensor for lactic acid detection is presented, with bioelectrodes composed of D-Lactate dehydrogenase immobilized on multi-walled carbon nanotubes using PDMS as a flexible substrate platform.

Additionally, in an innovative and recent study by Jeevarathinam et al. [34], it has been demonstrated that the combination of PDMS with polyethylene glycol (PEG) and ethyl cellulose (EC) nanoparticles enhances lactate sensors. Specifically, a highly sensitive and biocompatible phosphorescent oxygen sensor has been developed for continuous monitoring of lactate and glucose. The results show that EC nanoparticles, when conjugated with PDMS-PEG, are three times more sensitive and have an increased lifetime by 11 times between ambient and anoxic conditions, representing a significant advancement compared to previous studies that also employ this material.

### 3.4. Hydrogel

Hydrogels, hydrophilic polymeric networks with high water content, stand out as highly biocompatible biomaterials. Their mechanical properties resemble human cutaneous tissues, making them versatile platforms. Their biodegradability and ease of functionalization enhance target analyte specificity.

Despite characteristics such as self-healing, water absorption capacity, flexibility, and transparency, their inherent low conductivity limits their use. However, this is overcome by hybridizing with conductive materials and functionalizing with redox species and biomolecules. This biomaterial finds application in non-invasive extraction of biofluids (e.g., iontophoresis) and biorecognition, highlighting its role as transducers [20,35,36].

Other limitations associated with hydrogels include their high costs, low mechanical strength, and limited stability [36,37,38]. Their degradation over time and susceptibility to skin interferences, such as sweat and pH changes, compromise the accuracy of biosensors in measuring biomarkers. On the other hand, there are challenges related to their mechanical properties, critical for their use in clinical settings, where they must be flexible while maintaining structural integrity. Temperature also influences their durability: high temperatures soften them and make them more prone to damage, while low temperatures make them harder and more prone to fracture. These variations can affect their long-term performance in biosensors, limiting their applicability in clinical environments such as ICUs. Understanding these limitations and inherent challenges in using hydrogels is crucial for their effective application in specific environments. It is important to note that these challenges can be addressed through technological advancements and further research to ensure the efficacy and safety of these biosensors in critical clinical settings.

The study by Nagamine et al. [39] demonstrated the viability of a tactile electrochemical biosensor based on a hydrogel, extracting and detecting lactate through sweat non-invasively. De la Paz et al. [17] developed a soft, portable epidermal adhesive patch integrating a reverse iontophoresis system and an amperometric biosensor that allows for the collection of lactate from interstitial fluid and its subsequent analysis without any effort from the patient.

### 3.5. Liquid Metal Alloys and Ionic Liquids

Liquid metal alloys exhibit remarkable characteristics such as high conductivity, fluidity, flexibility, and infinite ductility, along with biocompatibility. They stand out for their patterning, printing, and self-healing capabilities. In their liquid state, they conform to the geometry of the container at room temperature, avoiding mechanical mismatches with human skin. Enhancing properties by adding nanomaterials and the popularity of gallium alloys expands their potential in various applications [40].

These materials act as circuits, flexible connectors, and conductors in the electrodes of deformable portable biosensors and patches, collecting physiological signals based on electrical potential difference and serving as conductive wires for connection. Additionally, they are used in portable biosensors to adhere to the human body without experiencing significant failures and, due to their deformability, allow for the precise detection of physiological signals and joint movements [31,40].

In the context of lactate monitoring, carbon nanotubes have emerged as a promising material in biosensors. The recent study by Lee et al. [41] focuses on developing an e-tattoo using gallium-based liquid metal particles decorated with platinum on carbon nanotubes. This configuration achieves intrinsic electrical conductivity and mechanical durability. The e-tattoo was characterized with key enzymes, such as glucose oxidase, alcohol oxidase, and lactate oxidase, enabling the detection of glucose, ethanol, and lactate. This innovative approach offers possibilities for more effective and specific real-time monitoring of lactate levels, opening new perspectives in biosensor research for clinical applications.

### 3.6. Nanomaterials

In this section, we highlight the significance of nanomaterials in enhancing the physical and mechanical properties of biosensors. Nanometric dimensions favor interaction with biomolecules, optimizing sensor response. Adaptability to patient movements is essential, and nanomaterials demonstrate high stretchability, flexibility, and low variability in resistance, making them ideal for implementation in these devices [42].

## 4. Non-Invasive Sensors for Lactate Monitoring

**Figure 2:**
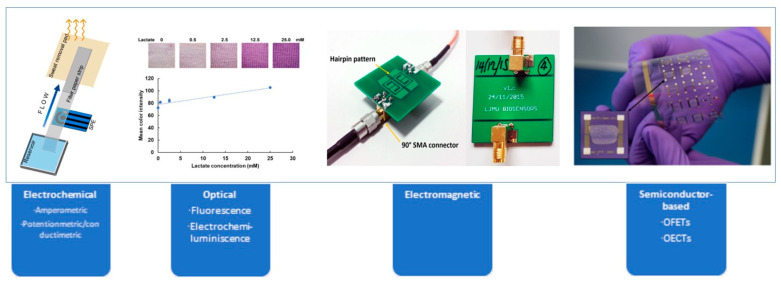
Overview of sensor technologies. Reprinted with permission from authors [43,44,45,46].

This section will discuss various sensor types used for lactate monitoring, including electrochemical, optical, electromagnetic, and semiconductor-based sensors. Figure 2 shows an overview of the different sensor types.

### 4.1. Electrochemical Sensors

Electrochemical sensors detect lactate through chemical reactions that generate electrical signals proportional to lactate concentrations. The response, based on a redox reaction, is linear with lactate under constant potential. Selectivity is enhanced by incorporating a biological recognition element into the sensor design, commonly using selective enzymes due to their biocatalytic properties and high specificity. These advancements offer promising prospects for precise lactate measurements [47,48].

Widely used in lactate measurement, their simplicity, versatility, and low cost are notable. They are distinguished by their rapid response, crucial for real-time monitoring. Their high sensitivity and selectivity to this metabolite allow for the detection of very low concentrations accurately. Additionally, these sensors are known for their excellent portability, which is particularly valuable for critical medical applications [47,49,50,51,52].

However, they typically have a short lifespan, necessitating frequent replacements [49]. They require a transcutaneous connection, posing additional challenges for clinical applications [53]. A significant limitation is the need to use biological elements in their design, which may be vulnerable to contamination and deterioration, negatively impacting measurement reliability. The interference from contaminating agents and chemical compounds in the biofluids analyzed can lead to signal reductions and decreased measurement accuracy [49].

#### 4.1.1. Technologies in Electrochemical Measurement

**Figure 3:**
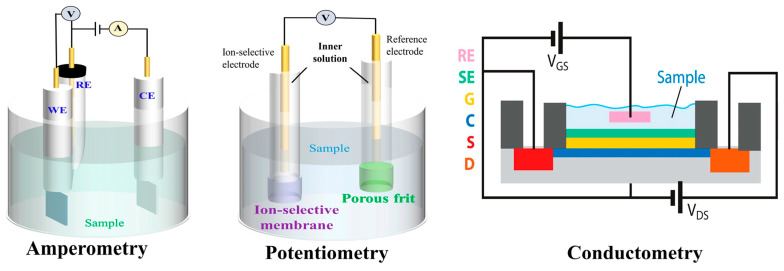
Schematic of the working principles for different types of electrochemical biosensors. Reprinted with permission from authors [20].

There are several technologies available for electrochemical measurement (see Figure 3). Firstly, amperometric sensors are most commonly used in lactate detection due to their simplicity and low cost. These sensors measure and monitor the electrical current between a working electrode and a reference electrode when a potential difference is applied. The current measured is directly related to the concentration of the electroactive species of interest, such as lactate. Amperometric sensors can detect changes in the metabolite’s concentration, related to its consumption or production in the sample. This allows for not only quantifying its concentration but also monitoring its variation over time [49,54].

Secondly, potentiometric and conductimetric techniques operate similarly to amperometric sensors. Instead of measuring the electrical current, the electrical potential difference is calculated. This difference is achieved through the coupling of an ion-selective membrane (ISM) to the working electrodes. The ISM is an essential component that allows the correlation between lactate activity and the recorded voltage. In this process, the ISM acts as a selective barrier that permits only lactate ions to pass through. The potential recorded at the ion-selective electrode (ISE) reflects the activity of these lactate ions, providing an accurate measure of the metabolite’s concentration in the sample [49,52]. Potentiometric sensors, though less common than amperometric ones, offer attractive advantages for implementation in clinical biosensors. They are known for their simplicity and robust design, aligning with amperometric sensors [52]. Their distinguishing feature is their low power consumption, operating with a polarization current as low as 10^−15^ A [52]. This low current minimizes voltage loss, known as ohmic drop, making them less susceptible to electrical interferences compared to amperometric sensors [52,55]. Additionally, potentiometry allows for the reduction of electrode size without compromising sensitivity, ideal for lactate biosensors that require miniaturization [52,56]. In summary, potentiometric sensors present key characteristics for clinical applications, offering energy efficiency and resistance to electrical interferences.

#### 4.1.2. Notable Materials in Electrochemical Measurement

In the realm of electrochemical sensors, several materials and techniques merit emphasis: electrode modification, selective membranes, nanomaterials, molecularly imprinted polymers, Prussian blue, and enzymes.

##### Electrode Modification

Electrode modification has seen significant advancements, as evidenced by Mengarda et al.’s study [57], particularly in the use of graphite pencil electrodes. Modification is achieved through an electropolymerization technique, depositing a layer of lactate-doped polypyrrole polymer on the electrode surface. This modification markedly enhances the electrode’s sensitivity and selectivity. The conductive nature of graphite provides a foundation for electrochemical detection, while the lactate-doped polypyrrole polymer modification facilitates a specific interaction with the analyte of interest, in this case, lactate. The doped polymer converts lactate concentration into a quantifiable electrical signal, thereby enabling the precise and sensitive detection of lactate in biological samples.

Recent advancements also include the implementation of solid contact electrodes, such as conducting polymers or carbon nanotubes, further enhancing the capabilities of electrochemical sensors, particularly potentiometric ones. These solid electrodes offer additional benefits like lower detection limits and miniaturization possibilities [49].

##### Selective Membranes

Selective membranes represent a significant advance in improving the performance and accuracy of these sensors. These membranes play a crucial role around the sensor electrode, acting as a barrier that permits controlled passage of lactate while filtering out interfering substances, leading to notable improvements in the accurate measurement of lactate in biological environments.

Weltin et al.’s article [58] highlights the importance of this selective membrane, which plays a fundamental role around the sensor electrode. It acts as a barrier allowing selective passage of lactate and rejecting other interfering substances, providing significant improvements in the accurate measurement of lactate in biological environments.

The selective membrane applied to the sensor electrodes is manufactured using specific techniques, such as the electropolymerization of 1,3-diaminobenzene. This membrane is designed to allow the controlled diffusion of lactate to the electrode while preventing interference from other chemical species present in the biological matrix.

Inclusion of this selective membrane enhances the lactate sensor’s specificity by minimizing responses to interferents like ascorbic acid, dopamine, or uric acid. This rejection of interferences is crucial for obtaining precise and reliable lactate measurements in complex and real-time environments.

##### Nanomaterials

Nanomaterials, with dimensions between 1 and 100 nanometers, play an essential role in lactate electrochemical sensors. Their small size imparts unique physicochemical properties, such as chemical stability, high electrical conductivity, and catalytic capability, enhancing electrochemical signals. Their biocompatibility makes them highly useful in biomedical applications, notably in biocompatible cellular uptake and ease of functionalization, providing new characteristics to these materials.

Nanomaterials on the electrode surface are crucial in modulating current flow, leading to significant improvements in the sensitivity, selectivity, reproducibility, and stability of these devices. They are also used as catalysts in biosensors requiring accelerated chemical reactions to determine the concentration of the monitored biomarker, yielding rapid responses [42,59,60].

The implementation of nanomaterials like graphene and carbon nanotubes is fundamental in immobilizing enzymes on electrodes, enhancing sensor stability. Similarly, these nanomaterials are used as redox mediators, serving as excellent catalysts for rapid biosensor responses, improving electrochemical detection. These materials are also applied in non-enzymatic biosensors, overcoming limitations of enzymatic biosensors through the use of metal nanoparticles mimicking enzymatic functions, and carbon nanotubes improving the sensor’s electronic properties [59].

Specifically, in potentiometric sensors, carbon nanotubes could be included in the contact electrode material, overcoming limitations and enhancing device performance [49]. Ibupoto et al. [61], developed a potentiometric biosensor for L-lactic acid detection using ZnO nanorods for enzymatic immobilization. This biosensor demonstrated a wide linear detection range, good sensitivity, and selectivity for L-lactic acid, highlighting the positive impact of nanomaterials on enhancing potentiometric sensor capabilities.

In specific applications like the real-time monitoring of epidermal sweat for lactate detection, metallic nanoparticles like gold in hedgehog form have been used to accelerate electron transfer and improve biosensor stability [62]. Similarly, Poletti et al. [43] employed graphene in an enzyme-based electrochemical device for monitoring glucose and lactate in sweat. In these applications, enzymes were anchored to nanomaterial platforms such as graphene oxide, while chitosan served as a medium for dispersing nanomaterials or nanocomposites, enabling stable deposition of bioreceptors. This demonstrates the versatility and effectiveness of nanomaterials in various biosensor applications.

Hashemzadeh et al. [63] conducted a study using a platinum electrode modified with a compound of reduced graphene oxide, carbon nanotubes, and gold nanoparticles for lactate detection, immobilizing the LOX enzyme. This amperometric biosensor exhibited a wide linear analytical range, high electrochemical sensitivity, and stability in environments with varying oxygen levels, underscoring the positive impact of nanomaterials on enhancing lactate detection in diverse environments.

Applications of nanomaterials in electrochemical sensors have shown significant improvements in the sensitivity, selectivity, stability, and overall performance of these devices, positioning them as essential components in the evolution of lactate detection technology.

##### Molecularly Imprinted Polymers (MIPs)

Molecularly imprinted polymers (MIPs) are non-enzymatic sensors designed to circumvent the drawbacks associated with the use of biological components in electrochemical sensor measurement technology.

Capable of electrocatalysis, MIPs achieve high selectivity and affinity in lactate detection. They are polymeric materials obtained through a novel 3D printing technology called Molecular Imprinting Technology (MIT), designed for selective recognition and binding to a specific analyte, making them robust materials for molecular recognition. Thanks to this property, they can mimic natural recognition entities like antibodies and are useful for separating and analyzing complex samples [64,65].

They stand out for their durability, maintaining optimal performance for several years. Moreover, these sensors act as artificial receptors, facilitating transduction and protecting against performance degradation caused by contaminants, pH variations, or temperature changes. Their versatility extends to functioning as biocatalysts, mimicking enzymatic function and broadening their potential applications [66].

In the research conducted by Mustafa et al. [67], a sensor was developed using molecularly imprinted polymers that are sensitive and selective for lactate detection, using methacrylic acid and ethylene glycol dimethacrylate as the functional monomer and crosslinking agent. Its potential as an artificial receptor for lactate molecules was observed, offering a good alternative with which to overcome the limitations of non-invasive detection based on enzymes and antibodies.

Despite MIPs’ advantages, limitations persist, such as low conductivity and sensitivity in detecting biomarkers in sweat. The introduction of conductive nanomaterials has improved conductivity and amplified signals. Additionally, nanomaterials have been applied to optimize the electrode’s surface and structure, increasing binding sites and enhancing biomarker detection sensitivity in sweat samples [65,68].

In the research line to improve the limitations of this material, Pereira et al. [69], developed a lactate sensor based on MIP, reduced graphene oxide (rGO), and gold nanoparticles (AuNPs). The results showed excellent sensor performance with high response and good stability, where MIP provides excellent selectivity towards lactate, AuNPs help improve conductivity, and rGO provides a high surface area. New materials offer exceptional selectivity and affinity towards analytes, ensuring reliable measurements. They stand out for their physical robustness, resistance to extreme conditions, and prolonged durability, surpassing enzymes. Affordably synthesizable, these materials exhibit an optimal performance for years [64,68].

##### Prussian Blue

Prussian Blue (PB), a dark blue pigment composed of FeO and Fe_2_O_3_ atoms linked to cyanide, is notable for its exceptional selectivity, sensitivity, biocompatibility, stability, and durability. Its primary attribute lies in its outstanding electrochemical catalysis capability [70,71]. PB has been demonstrated to provide an optimal electrocatalytic effect in the oxidation of H_2_O_2_, being three times more effective than regular platinum materials [49,70]. This advancement implies that sensors can operate with an electrical potential very close to zero, resulting in a very narrow working potential range where interferences are minimal [49,72,73].

A study by Gao et al. [74] highlights the viability and efficiency of a fully integrated, flexible sensor for the real-time monitoring of metabolites, such as lactate, in subjects’ sweat during physical activities. In this study, Prussian Blue was used as a redox mediator, demonstrating its efficacy in enhancing the performance of amperometric technology sensors.

Pleshakov et al. [75] developed electrochemical lactate biosensors based on Prussian Blue nanoparticles, immobilizing lactate oxidase via a coating method on the sensor surface with a mixture containing the enzyme, (3-aminopropyl) triethoxysilane, and isopropanol, showcasing its sensitivity and stability for application. Similarly, Vokhmyanina et al. [76] fabricated an electrochemical enzyme-based sensor for lactate monitoring, substituting the enzyme peroxidase with Prussian Blue at the working electrode.

##### Enzymes

Enzymes play a crucial role in generating quantifiable signals. Enzymatic sensors utilize L-lactate dehydrogenase (LDH) and L-lactate oxidase (LOD) as biorecognition elements.

LDH catalyzes the conversion of L-lactate to pyruvate and NADH, acting as an electron transporter between lactate and the electrode. This allows the oxidation of NADH on the electrode surface under an applied potential, generating a current directly proportional to the L-lactate concentration in the solution. Conversely, LOD catalyzes the oxidation of L-lactate to pyruvate in the presence of dissolved oxygen, generating hydrogen peroxide, an electrochemically active species.

Despite their effectiveness, LOD-based sensors can be prone to interferences due to the high oxidation potential required for hydrogen peroxide and fluctuations in oxygen concentration in the solution, impacting detection limits and practical application miniaturization. Continuous improvements in LDH-based biosensors have been achieved using various transducers and highly purified enzymes. These enzymatic biosensors provide an effective means of detecting lactate in biological solutions, enabling accurate and selective measurements.

Advancements in the integration of enzymes into electrochemical sensors have significantly contributed to the development of sensitive and specific devices for lactate detection in medical, sports, and health monitoring applications [51].

### 4.2. Optical Sensors

Optical sensors for lactate detection are based on measuring photons generated in enzymatic reactions on the sensor’s surface, eliminating the need for skin connections. Comprising a sensing layer, signal conversion device, and amplifier, these sensors use optical signals and fall into two main categories: electrochemiluminescence and fluorescence. These technologies are noted for overcoming interferences, reducing sample size requirements, and ensuring precise measurements. They exhibit high sensitivity for detecting substances in minute concentrations, offer rapid responses crucial for real-time monitoring, and their simple operation makes them attractive for diverse applications. The use of light transfer means that these optical sensors do not require physical connections through the skin, ensuring comfort compared to electrochemical methods. It is pertinent to emphasize their non-destructive operation, preserving the integrity of the analyzed samples [20,47,53,77,78].

Optical sensors, despite their benefits in lactate measurement, present challenges. Their manufacturing and maintenance are costly, and the fragility of optical components makes them susceptible to damage and complicates miniaturization. Interferences from ambient light and signal fluctuations due to external factors, such as temperature and pH, can compromise accuracy. The use of fluorescent dyes may result in photobleaching and affect biomolecule activity. These considerations are crucial in designing and employing optical sensors to ensure precise lactate measurements [47,51,79,80].

#### 4.2.1. Optical Sensor Technologies: Fluorescence and Electrochemiluminescence

**Figure 4:**
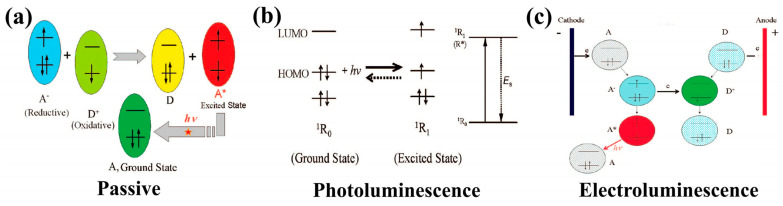
Schematic of the working principles for different types of optical biosensors: (**a**) Passive, (**b**) Photoluminescence, (**c**) Electroluminescence. Reprinted with permission from authors [20].

Figure 4 displays a summary of technologies involved in optical sensors. 

##### Fluorescence

Fluorescence-based optical sensors detect fluorescence generated by enzymatic reactions or specific probe interactions with lactate. Widely used to quantify lactate concentration in biological samples, they offer precise and specific measurements. Their versatility allows for lactate level detection across a broad range, valuable in clinical practice. Miniaturization and integration into systems such as chips facilitate adoption in point-of-care medical settings and real-time monitoring. However, they face challenges like rapid photobleaching of fluorescent dyes and potential loss of biomolecule activity due to dye conjugation [47,79,80].

Enzyme-based fluorescence sensors typically use lactate dehydrogenase and its coenzyme NAD, with the latter absorbing light at 340–360 nm and emitting intense fluorescence at 450–460 nm. The presence of lactate in the sample leads to its conversion to pyruvate by the enzyme, producing NADH as a byproduct. This process releases energy as fluorescence, which, when measured, determines the lactate concentration present in the sample. Higher lactate concentrations result in greater fluorescence intensity [79,81]. Non-enzymatic fluorescence sensors utilize specific probes that directly interact with lactate in the sample, generating a fluorescent signal corresponding to the lactate concentration [79].

In research by Groegel et al. [82], lactate measurement was conducted using a probe based on the fluorophore 4-amino-1,8-naphthalimide coupled with p-anisidine as a redox-active group for hydrogen peroxide detection. The oxidation of p-anisidine by hydrogen peroxide suppresses the photoinduced electron transfer process, increasing fluorescence intensity. This platform was used as a lactate biosensor with a detection limit of 164 nm, significantly lower than other commercial lactate assays [79]. These probes offer advantages such as greater stability and reduced susceptibility to interferences affecting enzymatic sensors. Additionally, eliminating the need for enzymes reduces the expenses associated with manufacturing and maintaining these sensors.

Furthermore, fluorescence sensors can be categorized based on their use of enzymes or non-enzymatic technologies and whether they employ labeling for measurement [20]. Labeled fluorescence sensors require an external light source to excite the labeling material and generate fluorescence. The emitted fluorescence intensity is directly related to the concentration of the specific analyte, varying as the analyte concentration changes. Non-labeled fluorescence sensors utilize the intrinsic fluorescence of certain substances or changes in the optical properties of sample components when interacting with an analyte like lactate. Among non-labeled sensors, colorimetric optical sensors stand out, based on detecting lactate in biological samples such as sweat through a visible color change in the device. This color change can be observed visually or compared with an incorporated color reference in the device. In some cases, this comparison can also be made using a camera or imaging system if optical sensors are included. The intensity or nature of the color change is directly related to the lactate concentration in the sample. This approach allows for rapid and real-time lactate measurement in a straightforward and affordable manner [83,84]. A study by Promphet et al. [44] describes developing a flexible device capable of measuring lactate and other biomarkers in sweat by incorporating colorimetric reagents that react with the analyte, resulting in an observable color change. Comparing the color change with a reference incorporated into the device enables quantitative lactate measurement.

##### Electrochemiluminescence

Electrochemiluminescence-based sensors generate photons as a result of the relaxation of excited molecules in an electrochemically initiated reaction. The emitted photon intensity is proportional to the concentration of one or more reactants involved in the electrochemical reaction. In lactate detection, when the metabolite is oxidized under the catalysis of immobilized enzymes such as lactate dehydrogenase and peroxidase using NAD+ as a coenzyme, electroactive H_2_O_2_ is produced. This H_2_O_2_ enhances the electrochemiluminescence of luminol, enabling its detection. These sensors are characterized by their light emission without the need for an external excitation source, as the energy is released during the chemical reaction in the presence of oxygen or during the oxidation of hydrogen peroxide [47,79].

Although chemiluminescence lacks high selectivity, it is distinguished by its simple and low-cost instrumentation, lower detection limits, a broad dynamic range, and a low signal-to-noise ratio [47,80,85]. It is also important to note that the efficiency of light emission in electrochemiluminescence sensors is affected as the pH decreases towards the neutral values necessary for optimal enzyme activity. To address this challenge, flow injection analysis systems have been developed, in which each enzyme-catalyzed reaction is conducted separately under optimal conditions to ensure ideal operation [79,86].

In a study by Martínez-Olmos et al. [85], an innovation in lactate detection using electrochemiluminescence was presented. A portable luminometer with single-use biosensors was developed, utilizing lactate oxidase and the electro-oxidation of luminol to measure lactate. This sensor offers portability, high sensitivity, and rapid data acquisition in 2.5 min, valuable in clinical applications. The electrochemiluminescence technique allows for precise lactate measurements at low concentrations with a detection limit of 2.4 µM and a reproducibility of 7–10%. This advancement makes lactate detection using this measurement technology more convenient and accurate in clinical contexts.

#### 4.2.2. Materials in Optical Sensors

Recent advancements in the field of optical sensors have spotlighted the use of metallic nanoparticles and quantum dots.

##### Metallic Nanoparticles

In recent years, the application of metallic nanoparticles to the development of optical biosensors has garnered significant research interest due to their unique optical properties. Their size and shape bestow upon them characteristics such as quantum effects and surface plasmon resonance, which enhance the detection of analytes like lactate. These nanoparticles exhibit exceptional fluorescent properties. The versatility of their absorption and emission bands allows for the tuning and control of these properties, aiding in the development of specific biosensors. Notably, these nanoparticles have the capacity to amplify optical signals, thereby enhancing the sensitivity of biosensors, as even minimal analyte concentrations can yield detectable signals. Additionally, their ability to emit or scatter light across various wavelengths enables the development of multiplexed biosensors, improving efficiency and reducing analysis times [87].

Current research continues to explore their use for lactate monitoring in optical biosensors, as evidenced by studies like that by Escalona-Villalpando et al. [88], where Fe_2_O_3_ and ZnO nanoparticles were synthesized as peroxidase mimetics for lactate quantification in a colorimetric lactate biosensor. These nanoparticles improved the stability and catalytic activity of LOX enzymes, yielding a more stable signal. Moreover, they exhibited high selectivity and a broad linear range, even outperforming commercial lactometers in lactate oxidation reaction response. In another study by Ma et al. [89], a cascaded ratiometric fluorescent sensor based on blue-emitting carbon quantum dots (CQDs) and silver nanoparticles (AgNPs) was designed. Lactate detection and quantification were achieved through designed mechanisms utilizing these three nanocompounds via oxidation reactions producing H_2_O_2_.

##### Quantum Dots (QDs)

Quantum dots have emerged as a pivotal component in optical sensors for lactate detection. These quantum dots possess photoluminescent properties, making them ideal for use as probes in lactate measurement. Their implementation in optical technology is predicated on their capacity for fluorescence and simultaneous reaction with specific enzymes like lactate oxidase. The conjugation of QDs with this enzyme allows for interaction with lactate in a sample, generating hydrogen peroxide (H_2_O_2_) as a byproduct, leading to a reduction in the fluorescence intensity of the QDs. The decrease in light emission from the QDs correlates directly with lactate concentration in the sample.

Studies by He et al. [90] and Yang et al. [91] exemplify the use of quantum dots in optical sensors for lactate detection, employing them in hydrogel matrices and hydrogel microspheres, respectively, with lactate oxidase enzyme. Both cases demonstrate how the reduction in quantum dots’ fluorescence in the presence of lactate enables rapid and accurate measurement of lactate levels, either visually or through devices like smartphones. Another study by Zhou et al. [92], conducted an assay to detect LDH activity in human serum samples using silicon quantum dots (SiQDs). These SiQDs demonstrated high selectivity, sensitivity, and rapid response in detecting LDH activity through changes in fluorescence intensity.

### 4.3. Electromagnetic Sensors

These sensors operate based on the interaction between emitted microwaves and the electromagnetic properties of the biological sample containing lactate. When microwaves interact with the sample, lactate polarizes in response to this electromagnetic field due to its polar nature. This polarization affects the conductivity and permittivity of the medium, which refers to the capacity to transmit an electric current and field, respectively. The functionality of these sensors hinges on detecting changes in permittivity induced by lactate presence. As lactate concentration in the analyzed sample increases, so does the permittivity of the medium proportionally. Quantifying these changes in permittivity enables the calculation of lactate concentration in the sample [45,79,93,94].

This measurement technology stands out due to its energy efficiency, operating at a very low power of typically 1 mW [79]. This energy efficiency, combined with its capability for high penetrance in analyzed fluids without the use of ionizing radiation and in a non-invasive manner, makes it particularly valuable in medical applications [45,93]. Another key aspect is its ability to provide real-time measurements, crucial in clinical environments. Recently, Mason et al. [45] developed a non-invasive microwave sensor capable of measuring blood lactate levels in real time. The sensor design allowed for effective microwave penetration, reducing interference and improving accuracy. The sensor was capable of measuring lactate levels in a range of 0–12 mM with a 13.4% error rate. Although its use was tested only in individuals during physical activity sessions, it was posited to provide relevant information in clinical practice. However, further development and research are required to determine its real efficacy in patients. Moreover, microwave sensors are attractive due to their lower cost compared to more traditional chemical measurement methods, making them accessible and economical [45,93]. Collectively, these advantages position microwave and electromagnetic measurement technology as a promising option for lactate measurement in clinical applications and potentially replacing other chemical measurement technologies in the future [79].

However, lactate measurement technologies based on microwaves present significant challenges that need to be addressed before determining their efficacy in clinical practice. One of the main limitations is their sensitivity and accuracy [45,94]. This is attributed to the lack of development of these types of sensors with the necessary precision for reliable clinical practice measurements. Therefore, further research and development, as well as clinical studies and real-patient testing, are required to validate their accuracy and reliability in medical applications. Another significant limitation of microwave-based sensors relates to their size and portability. Although some developed sensors are portable enough to be placed bedside, their design generally does not allow patients to move freely with these devices [45,79,94]. In most cases, electromagnetic sensors tend to be larger and less portable compared to those based on other measurement technologies like electrochemistry, making them less practical for clinical use where more compact and mobile devices are required.

#### 4.3.1. Technologies in Electromagnetic Sensors

There are no notable technologies in electromagnetic measurements.

#### 4.3.2. Materials in Electromagnetic Sensors

Recent research has focused on enhancing the sensitivity and accuracy of electromagnetic sensors. This has involved the exploration of metamaterials, materials exhibiting unusual electromagnetic properties aimed at optimizing sensor detection [95]. The primary advantage of using metamaterials in these technologies lies in their ability to precisely control the emitted electromagnetic waves. Metamaterials enable the adjustment and direction of waves so that they are highly sensitive to changes in medium properties, such as lactate concentration in a sample [94,95].

### 4.4. Semiconductor-Based Sensors

Lactate sensors employing semiconductor measurement technologies, through either organic field-effect transistors (OFETs) or organic electrochemical transistors (OECTs), operate on a general working principle. These devices exploit the ability of organic semiconductor materials to alter their electrical conductivity in response to external stimuli, here being the presence of lactate in a sample. This principle enables lactate detection and quantification by monitoring changes in the semiconductor material’s electrical conductivity [20,95,96,97,98,99,100,101].

Semiconductor-based sensors are typically flexible and adaptable, lending themselves to various applications. They also function at low voltage, contributing to energy efficiency and savings. Notably, some semiconductor sensor variants can not only detect lactate levels but also amplify the signal, which is advantageous in applications requiring rapid response and low-concentration detection. This signal amplification makes them promising for clinical and medical uses [20,95,96,97,98,99,100,101].

The main limitation of semiconductor-based sensors lies in their reliance on still-developing technologies. The reliability and accuracy of these sensors necessitate more detailed and specific research in clinical contexts to validate their reliability, along with continual improvements. Additionally, consistent calibration is crucial to ensure accurate measurements. Another significant limitation is their high sensitivity to environmental factors like temperature and humidity, which can affect measurement stability and accuracy [20,95,96,97,98,99,101].

#### 4.4.1. Semiconductor Sensor Technologies

##### OFETs

Lactate sensors based on OFETs typically use an enzyme, commonly lactate oxidase, for lactate detection [98,99,101]. When a lactate-containing sample is introduced to the OFET sensor, the enzyme catalyzes the conversion of lactate to pyruvate, generating electrons as a byproduct. The presence of lactate in the sample alters the electric current through the OFET, proportional to the lactate concentration in the analyzed sample, thus enabling metabolite quantification [99,100,101].

OFET sensors offer several advantages, including high flexibility, portability, and cost-effectiveness [98,100,101]. They are capable of rapidly responding to lactate concentrations, showing promise for clinical practice [99,101]. However, this measurement technology also has limitations, including the need for constant calibration to ensure accurate measurements and high sensitivity to temperature and humidity [99,100,101]. Despite their advantages, OFET sensors are still in the early development stages, limiting their availability and application in medical contexts.

##### Organic Electrochemical Transistors

Sensors utilizing OECTs of the *p*-type follow the common operating principle of all OECTs. This principle is based on adjusting the electrical conductivity of organic semiconductor materials in response to external stimuli, such as the presence of lactate molecules in a sample [96]. This technology allows for the detection and quantification of specific substances depending on how they influence the electrical conductivity of certain organic materials. Applying an electrical potential between the electrodes of a *p*-type OECT generates an electric field in an organic semiconductor material, like polypyrrole (PPy) or polyaniline (PANI) [96]. This electric field affects the movement of charge carriers, like electrons or holes, through the material, altering the semiconductor’s conductivity. The resulting change in conductivity correlates with the density and mobility of the charge carriers, influenced by the interaction with lactate molecules. *p*-type OECTs have the advantage of enabling flexible sensor designs, operating at low voltages, and having a simple and economical preparation process [20,97]. They also serve dual functions, not only detecting lactate molecules but also amplifying the corresponding signal, making them valuable for lactate detection [97].

Conversely, *n*-type OECT sensors operate on the same principle as *p*-type OECTs but use *n*-type organic semiconductor materials such as naphthalene-1,4,5,8-tetracarboxylic diimide (NDI) conjugated polymers for high sensitivity and wide-range concentration measurements [102]. Pappa et al. [103] developed an *n*-type OECT lactate sensor with high sensitivity and a broad concentration range, representing a significant advancement in metabolite detection using semiconductor sensors [20]. Additionally, Scheiblin et al. [46] presented a notable innovation in addressing the clinical applicability limitation of most lactate sensors incorporating OECTs. This innovation involved using a solid-state electrolyte in OECTs instead of traditional liquid solutions, essential for broader clinical applicability and practical lactate detection contexts.

#### 4.4.2. Materials in Semiconductor-Based Sensors

##### Carbon Nanotubes

Studies have shown that incorporating carbon nanotubes into the measurement structure of OFET-based sensors significantly enhances their sensitivity and efficiency [98,100]. Carbon nanotubes increase the electrode’s surface charge in the OFET sensor due to their high conductivity and specific surface area, leading to increased lactate detection sensitivity. These nanostructures also act as redox mediators by facilitating electron transfer between the enzyme and the electrode, thus improving the electrochemical detection efficiency of the metabolite. Moreover, carbon nanotubes provide an effective platform for enzyme immobilization in OFET sensors, ensuring sensor stability [98,100,101].

##### Metal Nanoparticles

Significant advancements have been made in semiconductor-based lactate sensor development with the application of metal nanoparticles. Gualandi et al. [97], focused on *p*-type OECT semiconductor sensors, with the developed device demonstrating notable improvements in measurement sensitivity and extremely low lactate concentration detection. Optimizing these sensors was achieved by modifying their electrodes with platinum nanoparticles and a chitosan solution. The inclusion of platinum nanoparticles played a crucial role, not only reducing the detection limit but also significantly enhancing sensor sensitivity. This improvement facilitated accurate and reliable lactate detection in samples, essential for clinical monitoring and biomedical applications.

##### Conductive Polymers

Conductive polymers represent a broad category of polymeric materials capable of conducting electricity. These polymers, a type of organic materials, are recognized for their unique electrical and optical properties, akin to inorganic semiconductors and metals. Their flexibility, elasticity, and biocompatibility make them versatile elements in various applications [20,104,105,106]. 

In a study by Pappa et al. [103], a focus was placed on developing a portable lactate sensor based on OECT. This sensor exhibited an effective detection range of 0.1–2 × 10^−3^ M. The approach involved combining PEDOT: PSS with polyvinyl alcohol (PVA), applied to the fabrication of the *p*-type OECT involved in lactate measurement. Implementing this polymer as a platform demonstrated an improvement in ion transport, resulting in a rapid, selective, and sensitive metabolic capacity for lactate detection.

## 5. Discussion

In evaluating the application of biosensor technology within the ICU context, critical assessment criteria such as efficacy in continuous, real-time lactate monitoring, cost-effectiveness, simplicity, and portability must be considered. A review of four different technologies reveals that each offers significant benefits but also has limitations that require careful consideration (see Table 1).

The electrochemical system, despite its short lifespan, currently yields the best results, meeting essential criteria like rapid response, high sensitivity, versatility, and portability for ICU application. It is cost-effective and simple to manufacture. The optical sensor, while more expensive and challenging to maintain, performs well and offers superior data collection compared to the electrochemical sensor. However, it faces challenges in miniaturization. Electromagnetic sensors represent an innovative but developing measurement technology, characterized by low sensitivity, limited portability, and size. Semiconductor-based sensors are still under research, and while their limitations might be overcome in future years, constant calibrations could increase the workload for healthcare professionals and lead to inaccuracies in measurements. In the short term, both electromagnetic and semiconductor technologies require further development to reach maturity.

Additionally, a comparative analysis of the analytical features of the lactate sensor is presented (see Table 2).

### Regulatory Considerations, Ethics, and Barriers to Adoption

This article does not address the potential for the development and marketing of the mentioned measurement methods. Nevertheless, should their industrial development and subsequent commercialization proceed, compliance with the current regulations of the applicable geographical area will be ensured, with special attention paid to European legislation.

In the scenario where a specific device is developed, once validated in a laboratory, it must be subject to clinical studies. These studies typically include initial phases with animals and, upon confirmation of their utility and safety, with healthy volunteers and patients. To commence, approval from the Ethics Committee for Research and Animal Welfare or the Drug Research Ethics Committee is required in each instance.

Considering that these are non-invasive measurement devices classified as class I by the Spanish Agency for Medicines and Health Products, it is conceivable that the Drug Research Ethics Committee may expedite approval, potentially waiving the preliminary trial on animals. Despite this, even for non-invasive devices (class I), the procurement of clinical trial liability insurance is necessary.

Regarding ethical considerations, we must acknowledge that, during clinical studies, it is crucial to provide patients with comprehensive and understandable information about the utilized technology, obtaining their informed consent prior to implementation. Once authorized by regulatory bodies, patients’ implied consent will suffice unless explicitly stated otherwise by the regulatory agencies.

It must be ensured that the implementation of this technology does not create disparities in access to medical care, striving to make it available to all patients, regardless of their socioeconomic status.

As barriers to the adoption of the technology, we must consider the economic cost, personnel training, and resistance to change.

This topic can be expanded upon in Supplementary Document S1 (see Document S1), which, among other things, references the minimum standards to consider.

## 6. Conclusions and Future Prospects

This review has highlighted the advancements in emerging technologies and biosensor development for lactate monitoring, showcasing significant progress with revolutionary potential in clinical practice. These biosensors offer the possibility of non-invasive, real-time information gathering, providing healthcare professionals with tools that enhance decision-making and contribute to more personalized, predictive, preventive, participative, and population-based medicine. The integration of devices based on innovative technologies, such as microwave and semiconductor technologies, along with continual refinement and improvements in optical and electrochemical measurement technologies, has added further relevance to this field. Furthermore, significant materials, including carbon nanotubes, metallic nanoparticles, and flexible substrates, play a crucial role in enhancing the detection sensitivity, biocompatibility, and adaptability of these biosensors.

However, significant challenges persist in this domain. Most research and development of these technologies have not yet reached the commercial stage and have not been tested in clinical environments, raising questions about their validity and utility in clinical practice. Currently, the only proven and functional method is the electrochemical sensor, but in the short term, the optical sensor appears to be promising. In the longer term, semiconductor-based technologies warrant consideration as they develop. The complex nature of these technologies necessitates specific clinical research to validate and ensure their accuracy, reliability, and feasibility in medical settings.

In designing an IoMT sensor for continuous lactate monitoring in the ICU, advancements in optical sensors should be considered, aiming for a wireless sensor with a long lifespan in terms of both sensing and power supply. Hence, efforts should focus on improving optical sensors to mitigate the short lifespan of electrochemical sensors and on advancing systems that provide power to these sensors using methods like harnessing the body’s own energy or other sources.

## Figures and Tables

**Table 1:** Comparative analysis of lactate sensor characteristics (2024).

Evaluation Criteria	Electrochemical	Optical	Electromagnetic	Semiconductors
Technological Maturity	Very High	Medium	Low	Low
Lifespan	Short	Long	Long	Long
Versatility	Yes	Yes	N/A	N/A
Portability	Simple	Difficult	Limited	Under Research
Simplicity	Simple	Complex/fragile	Very difficult	Under Research
Cost	Inexpensive	Expensive	N/A	N/A
Efficacy in Continuous Monitoring	High performance	High performance	Low sensitivity and precision	Under Research for potential limitations

**Table 2:** Comparative analysis of lactate sensor analytical characteristics (2024).

Sensor Type	Electrochemical	Optical	Electromagnetic	Semiconductors
Linear Range	Wide	Wide	Narrow	Wide in n-type OECTs
LOD	Very low	Very low	Under Research	N/A
Detection Method	Based on chemical reactions generating electrical signals proportional to lactate concentrations	Based on photons generated in enzymatic reactions on the sensor’s surface	Based on the interaction between emitted microwaves and sample containing lactate	Based on organic semiconductor materials that change their conductivity in response to the presence of lactate
Rapid Response	Yes	Yes	Yes	Yes in OFETs
Sensitivity	Good	Good	Low	N/A

## Supplementary Materials

The following supporting information can be downloaded at: https://www.mdpi.com/article/10.3390/bios14030148/s1, Document S1: Regulatory Considerations, Ethics, and Barriers to Adoption.
