# Supplementary material for: Continuous and Non-Invasive Lactate Monitoring Techniques in Critical Care Patients
Source: Biosensors (Basel). 2024 Mar 18;14(3):148. doi: 10.3390/bios14030148 (PMC10968200; doi:10.3390/bios14030148)
Supplement: Supplementary file 1 [file biosensors-14-00148-s001.zip › biosensors-2886961-supplementary.pdf]

Regulatory Considerations, Ethics, and Barriers to Adoption

Regulatory Considerations:

This article does not contemplate the development and commercialization of the described measurement methods. However, if the industrial development and subsequent marketing of these are pursued, it will adhere to the relevant regulations of the geographical area in question, with particular attention to European legislation. In the event of their development, the specific device, once laboratory-validated, must undergo clinical trials. These trials typically commence with initial animal phases and, following confirmation of utility and safety, proceed with healthy volunteers and patients. Conducting these requires prior authorization from the Research Ethics and Animal Welfare Committee or the Drug Research Ethics Committee in each case.

As these are non-invasive measuring devices classified as Class I by the Spanish Agency for Medicines and Health Products (Agencia Española de Medicamentos y Productos Sanitarios, AEMPS), and pursuant to Royal Decree 1591/2009 of October 16th, which regulates health products among other provisions, approval from the ethics committee may be facilitated, potentially even waiving the preliminary animal trial. Nevertheless, for such non-invasive devices (Class I), Clinical Trials Civil Liability Insurance is mandatory, in accordance with Article 61 of the Legislative Royal Decree 1/2015 of July 24th, which approves the consolidated text of the Law on Guarantees and Rational Use of Medicines and Health Products, and as defined in the risks and terms of Articles 9 and 10 of Royal Decree 1090/2015 of December 4th, regulating clinical trials with medicines, Ethics Committees for Drug Research, and the Spanish Registry of Clinical Studies.

Upon achieving positive results in clinical trials, communication will proceed with the pertinent regulatory bodies, such as the AEMPS and the European Medicines Agency (EMA). The manufacture of devices must meet strict quality and safety standards to ensure efficacy and precision in critical clinical settings. These bodies will determine the safety and indications for the devices or measuring methods based on study outcomes and compliance with quality standards in device construction.

Given the sensitive nature of health data, Article 9 of the GDPR (General Data Protection Regulation), compliance with privacy and data protection regulations is imperative according to the Organic Law 3/2018 of December 5th, on Personal Data Protection and Guarantee of Digital Rights (Ley Orgánica de Protección de Datos Personales y Garantía de los Derechos Digitales or LOPD-GDD) in Spain and the General Data Protection Regulation (GDPR) of the European Parliament and of the Council of April 27, 2016.

Ethical Considerations:

During clinical trials, it is vital to provide patients with comprehensive and comprehensible information about the employed technology, obtaining their informed consent before implementation. Once regulatory bodies grant approval, the patients' tacit consent will suffice unless regulatory agencies specify otherwise.

Equity in Access:

It is essential to ensure that the deployment of this technology does not create disparities in access to medical care, aiming to make it available to all patients, regardless of their socioeconomic status.

<u>Barriers to Adoption:</u>

Economic Cost:

Conducting clinical trials entails significant economic expenses, potentially necessitating research grants or commercial partnership involvement. In addition, device validation and certification involve substantial financial outlays, as does the device's construction.

Personnel Training:

Medical staff must receive proper training to utilize and interpret the data provided by these sensors, which may demand substantial time and resources.

Resistance to Change:

The introduction of new technologies often encounters resistance from medical personnel and patients, which can hinder widespread adoption.

By addressing these regulatory and ethical considerations, we aim to establish a robust framework for the implementation of non-invasive sensors intended for continuous lactate monitoring in critical care settings.

The minimum legislation to consider, which will have a direct or indirect impact in the event of proceeding with the development of the device presented in this article, includes:

- Law 29/2006 of July 26th, on guarantees and rational use of medicines and health products.

- Legislative Royal Decree 1/2015 of July 24th, approving the consolidated text of the Law on Guarantees and Rational Use of Medicines and Health Products.

- General Health Law 14/1986 of April 25th.

- Law 16/2003 of May 28th, on cohesion and quality of the National Health System.

- Law 24/2015 of July 24th, on Patents.

- Law 22/1994 of July 6th, on liability for defective products (Ley de Responsabilidad Civil por los Daños causados por Productos defectuosos, LRCDP), adapting the Spanish system to Directive 85/374/CEE of July 25th, and its amendment by Directive 1999/34/CE of May 1th 1999.

- Royal Decree 316/2017 of March 31st, approving the Regulation for the execution of the Patent Law 24/2015 of July 24th.

- Royal Decree 1345/2007 of October 11th, which regulates the authorization, registration, and dispensing conditions for industrially manufactured human use medicines.

- Royal Decree 782/2013 of October 11th, concerning the distribution of human use medicines.

- Royal Decree 1907/1996 of August 2nd, on advertising and commercial promotion of products, activities, or services with a purported health purpose.

- Royal Decree 824/2010 of June 25th, which regulates pharmaceutical laboratories, manufacturers of active pharmaceutical ingredients, and the foreign trade of medicines and medicines under investigation.

- Royal Decree 177/2014 of March 21st, which establishes the system of reference pricing and homogeneous drug groupings within the National Health System, and certain information systems regarding the financing and pricing of medicines and health products.

- Royal Decree 870/2013 of November 8th, which regulates the remote public sale via websites of non-prescription human use medicines.

- Royal Decree 577/2013 of July 26th, which regulates the pharmacovigilance of human use medicines.

- Royal Decree 1416/1994 of June 25th, which regulates the advertising of human use medicines.

- Royal Decree 1015/2009 of June 19th, which regulates the availability of medicines in special situations.

- Royal Decree 1785/2000 of October 27th, on the intracommunity circulation of human use medicines.

- Royal Decree 954/2015 of October 23rd, which regulates the indication, use, and authorization of dispensation of medicines and sanitary products for human use by nurses.

- Royal Decree 192/2023 of March 21st, which regulates sanitary products.

Regarding technological norms, at minimum standard UNE 60601, CE marking, among others, will be considered.
